# Using Plasma Etching to Access the Polymer Density Distribution and Diffusivity of Gel Particles

**DOI:** 10.3390/polym13152537

**Published:** 2021-07-31

**Authors:** Ivan J. Suarez, Benjamin Sierra-Martin, Antonio Fernandez-Barbero

**Affiliations:** 1 NanoLab, Department of Chemistry and Physics, University of Almeria, 04120 Almeria, Spain; isuarez@ual.es (I.J.S.); bsierra@ual.es (B.S.-M.); 2 Institute of Applied Chemical Sciences, Universidad Autonoma de Chile, Santiago 7500138, Chile

**Keywords:** pNIPAM, minigel, polymer density, diffusion, plasma etching

## Abstract

In this paper we examine the polymer density distribution of gel particles and its effect on solvent diffusivity through the polymer network. In order to access the inner particle regions, external polymer layers were removed by plasma etching, thus reducing them from the outside. Higher polymer densities after erosion showed internal heterogeneity, with the density increasing towards the center of the particles. An exponential decay polymer density model is proposed, and the spatial relaxation length measured. The diffusion of solvent through the particles, before and after the plasma oxidation, revealed a correlation between the diffusion coefficient and the internal density.

## 1. Introduction

Hydrogels are 3D polymer networks that absorb or expel water in response to environmental conditions. This is a direct consequence of transitions between equilibrium states resulting from the competition between molecular interactions and structural elastic contributions. Temperature, pH, ionic concentration and photo-irradiation are typical parameters triggering these transitions [1,2,3,4]. Hydrogels at equilibrium are described by the Flory-Rehner theory [5], which considers the balance between osmotic pressure due to polymer-solvent mixing and the elastic pressure due to the cross-links. The polymer network is described as a homogeneous mesh in which random microscopic structural fluctuations blur within average values describing the global system. However, polymer gels often exhibit large spatial density fluctuations that affect their global elastic features. For this reason, a considerable effort has then been made to gain insight into the underlying internal morphology of hydrogel particles [6,7].

The first research concerning inhomogeneous polymer distribution of microgels was reported by Wu et al. [8]. They tracked the synthesis of poly(N-isopropylacrylamide) particles (pNIPAM) in the presence of *N,N′*-methylene bisacrylamide (BIS), used as a cross-linker, and observed faster consumption of the cross-linker compared to the main monomer NIPAM. In fact, to obtain homogeneous cross-linker distribution, it was required to feed the monomer and cross-linker in a well-controlled way [9]. Scattering studies confirmed the inhomogeneous structure of microgels and its dependence on the degree of cross-linking [10]. Varga et al. found that highly cross-linked microgels exhibit a Gaussian segment density distribution in their swollen state, while with decreasing cross-linking, microgels are better described as core/shell structures of highly cross-linked cores surrounded by a shell of polymer chains.

Small angle neutron scattering (SANS) has been used for the characterization of the internal structure of pNIPAM particles as a function of temperature [11,12,13,14,15]. Fernandez-Barbero et al. observed a transition from Porod scattering for collapsed particles to a combination of Ornstein-Zernike scattering for swollen particles [13]. A core-shell form factor model was proposed with uniform density at the microgel core and a gradual decaying density toward the outer border. These inhomogeneities influence the microgel ability to swell and shrink. Particles do not only expand outwards during swelling but also inwards, thus collapsing the highly cross-linked core. This scenario was checked by monitoring the correlation lengths of inner and external domains with polymer expansion. SANS has also been used to describe the evolution of the morphology of biocompatible microgel particles based on ethylene glycol [16]. In order to study the effect of the irregular polymer distribution on the global behavior of hydrogels, Di Lorenzo et al. synthetized gels with defined extents of nanostructural heterogeneities [17]. The local microscopic Young’s modulus, determined by atomic force microscopy (AFM), was compared to the macroscopic elastic moduli from shear rheology. They found that the moduli are progressively smaller if the length scale of the probed gel region exceeds the size of the purposely-imparted polymer network heterogeneities. This finding was explained by the non-affine deformation of densely cross-linked polymer domains in the heterogeneous gels.

Inhomogeneous polymer density distributions have also been confirmed for vinylpyridine pH-sensitive microgels [18]. The standard Mie’s theory for homogeneous spherical particles was not enough to explain static light scattering experiments, even for particles completely collapsed. In addition, a model based on coils failed, particularly at a high swelling ratio where completely extended particles should behave as polymer coils. As a third approximation, a decaying exponential density distribution from the particle center was considered, reaching a good fit. Complex super-resolution microscopy was also employed to estimate density distribution [19]. The authors obtained good results by considering a decaying erfc profile for the density distribution.

In the present research, we studied polymer density distribution into minigels, which are gel particles in the range of several tens of micrometers. Different-sized pNIPAM minigels (~μm) were eroded from the outside by applying oxidizing plasma. In each run, a similar amount of mass was removed from each particle, since the same plasma dose was supplied to all particles. However, the particle volume reduction in each run depended on the original size of each particle and, consequently, shallower or deeper polymer shells were removed from each particle. We propose a decreasing exponential polymer density distribution for the minigels, which is high consistency with the results. Interestingly, a correlation was established among this polymer distribution and the solvent diffusivity across the polymer network during particle swelling. The study into this length-scale may add more universality to the knowledge of the natural inhomogeneity of hydrogels. As an application, the experimental method developed in this paper can be used for tuning the diffusivity of gel particles coming from a unique batch (which has cross-linking degree established by synthesis).

## 2. Theory

### 2.1. Gels at Equilibrium

Minigels belong to the family of polymer gels and, similar to other relatives, they reach thermodynamic equilibrium when the solvent chemical potential is equal inside and outside the gel. The osmotic pressure is then balanced ($\pi_{total}=0$) and no net transfer of solvent to and from the bulk takes place. For nonionic polymers, two main contributions establish the equilibrium, both described in the Flory-Rehner theory [20,21,22]. In the first, a mixing term (π*_m_*), is expressed in terms of the polymer solubility and accounts for the interaction between the polymer and the solvent molecules. The second contribution, the elastic term (π*_e_*), contains the elasticity of the polymer network. The elastic contribution is always involved in shrinking the gel, while the mixing contribution is of an ambivalent nature, depending on the interaction between the polymer and the solvent molecules. High polymer solubility leads to polymer swelling, whereas low solubility contributes to de-swelling. This ambivalence is determined by the Flory parameter, χ, around 0.5. The mixing osmotic pressure, $\pi_{m}=-\left(N_{a}kT/\nu_{s}\right)\left[\Phi +\ln\left(1-\Phi \right)+{\chi \Phi }^{2}\right]$, depends on both the polymer volume fraction, Φ, and the temperature, *T,* with *N_a_* and *k* being Avogadro’s number and Boltzmann’s constant, respectively. $\nu_{s}$ stands for the solvent molar volume. As mentioned above, the mixing term contributes to swelling or de-swelling, depending on the value of χ. For high polymer solubility (χ < 0.5), the mixing osmotic pressure increases with Φ, always favoring gel swelling. At low polymer solubility (χ > 0.5), the effect of the mixing contribution depends on the polymer density; the gel tends to swell for high polymer concentrations and to collapse for even higher Φ values. The network elasticity always contributes to gel de-swelling. It is set only by the network cross-link density *N_c_* (the network’s number of chains, which is assumed constant in the first approximation, along the whole network with homogenous density distribution) and modulated by the polymer volume fraction Φ, as $\pi_{e}=\left(N_{c}kT/V_{o}\right)\left[\frac{\Phi }{2\Phi_{0}}-{\left(\frac{\Phi }{\Phi_{0}}\right)}^{1/3}\right]$, with $\Phi_{0}$ being the volume fraction for the collapsed state. Both contributions do not work independently. When environmental changes (temperature or solvent (*T*, χ, $\nu_{s}$)) push the system out of equilibrium, it evolves to recover a new equilibrium state compatible with the settled conditions. The gel volume fraction (Φ) changes, thus tuning both contributions towards the new state established under the condition $\pi_{total}=\pi_{m}+\pi_{e}=0$. This rearrangement is the mechanism responsible for polymer gel swelling.

### 2.2. Evolution to the Equilibrium

A good approximation to describe the kinetics of polymer swelling was developed by Tanaka et al. [23]. The model describes the displacement of a point on the network from its final equilibrium location by a position vector $\vec{u}$(*t*). It determines the kinetics of swelling under the condition $\underset{t\to \infty }{\lim}\vec{u}\left(t\right)=0$. The equation of motion is given by Newton’s second law for a continuum medium, assuming the absence of acceleration of the network [12] $f\frac{\partial \vec{u}}{\partial t}=\nabla \cdot \overset{̿}{\sigma }$, where *f* is the solvent-network friction coefficient and $\overset{̿}{\sigma }$ is the stress tensor, $\sigma_{ik}=K\nabla \;\vec{u}\delta_{ik}+2µ\left(u_{ik}-\frac{\nabla \cdot \vec{u}\delta_{ik}}{3}\right)$. *K* and μ represent the network bulk and shear modulus, respectively, and $u_{ik}$ accounts for the cross derivatives, $u_{ik}\;\equiv \;\frac{1}{2}\left(\frac{\partial u_{k}}{\partial x_{i}}+\frac{\partial u_{i}}{\partial x_{k}}\right)$. This description establishes that the friction exerted by the liquid on the network balances the forces associated with the internal stresses (produced by changes of volume and shear deformations).

For spherically-symmetrical swelling, the general equation leads to a Fick-like diffusion equation for the position vector, $\vec{u}$(*t*):

(1) $$\frac{\partial \vec{u}}{\partial t}=D\cdot \frac{\partial }{\partial r}\left\{\frac{1}{r^{2}}\cdot \left[\frac{\partial }{\partial r}\left(r^{2}\vec{u}\right)\right]\right\}$$

with $D=\frac{\left(K+4µ/3\right)}{f}$ being the polymer-network diffusion coefficient [24]. The equation is solved by Fourier expansion, leading to an analytical expression for the size of a polymer spherical object as a function of the time [11]:

(2) $$r\left(t\right)=r_{f}-\left(\frac{6}{\pi^{2}}\right)\left(r_{f}-r_{i}\right)\overset{\infty }{\underset{n=1}{\sum }}\frac{\exp\left(-n^{2}\frac{t}{\tau }\right)}{n^{2}}$$

where *r*(*t*) represents the particle radius at a certain time, and *r_i_* and *r_f_* the initial al final particle radii, respectively. The time-scale *τ* is set by the equilibrium state (characterized by the final particle size, *r_f_*), and the ability of the polymer-network to spread through the liquid during the swelling (characterized by its time-averaged collective diffusion coefficient).

(3) $$\tau \;\left(r_{f},D\right)=\;\frac{r_{f}^{2}}{\pi^{2}\cdot D}$$

## 3. Materials and Methods

### 3.1. Synthesis of Minigels

Experiments were performed with cross-linked pNIPAM particles dispersed in water. They expanded below the critical temperature of 32 °C and collapsed at higher temperature. pNIPAM particles with 3 wt% cross-link concentration were prepared by inverse polymerization according to the method described by Suarez et al. [25]. NIPAM (Aldrich, 5.005 g), BIS (Aldrich, 0.155 g), and potassium persulfate (Aldrich, 0.352 g), were used as the main monomer, cross-linker and reaction initiator, respectively. Reagents were dissolved at room temperature in 40 mL of deionized water (below 1 µS/cm) into a 500 mL cylindrical round-bottom glass flask containing n-heptane (Aldrich, 200 mL) with the nonionic surfactant Hypermer B246 (UNIQEMA, 1.034 g). All reagents were used without further purification. In order to emulsify the water phase into the organic phase, the mixture was stirred at room temperature using a Heidolph-RZR2021 mechanical stirrer at 600 rpm with a single Teflon paddle. Stirring was then slowed down to 400 rpm after 1 h. The polymerization (started by setting the temperature to 70 °C) was kept for 12 h before sedimentation. The surfactant was then removed by rinsing five times with pure n-heptane. After dilution with 200 mL of deionized water, the organic phase was removed with a rotary-evaporator at 50 °C. The particles in the aqueous phase were left behind and organic traces removed by rinsing with deionized water.

### 3.2. Polymer Oxidation

To make visible the polymer density distribution at the interior of the gel particles, the external polymer was eroded by applying He:O_2_ plasma onto a collection of different-sized particles previously deposited on a glass slide (erosion by oxidation). Oxygen produces functional groups on the polymer with the formation of low-molecular-weight products. Excited CN, CH, HCHO, CH_3_O, C_3_H_5_, C_4_H_2_^+^ and CHO play a dominant role in the pNIPAM decomposition [26]. An Atomflo 250 system (Surfx™ Technologies LLC) generated the atmospheric plasma. A He:O_2_ mixture (30 L/min: 0.5 L/min) was injected into a 2.54 cm diameter plasma torch (AH-250D, Surfx™ Technologies LLC) and excited by 150 Watt 27.12 MHz RF. This frequency allowed us to operate efficiently with plasma at low temperature and pressure, which avoided any damage to the samples. Moreover, the plasma was guided directly to the minigel particles along a straight path by using a plasma torch assembled in a robotic arm. Plasma was applied at 5 mm/s constant sweeping speed and 1 mm torch-sample gap. At this speed, the plasma erosion rate calculated with the particle size before and after plasma was about 4.77 × 10^−9^ g/cm^3^ (the minigel density is considered to be 1.1 g/cm^3^). Particles for swelling experiments were selected from a narrow central region of the sweeping path to guarantee the same exposure time over all particles.

### 3.3. Particle Size-Distribution

**Figure 1:**
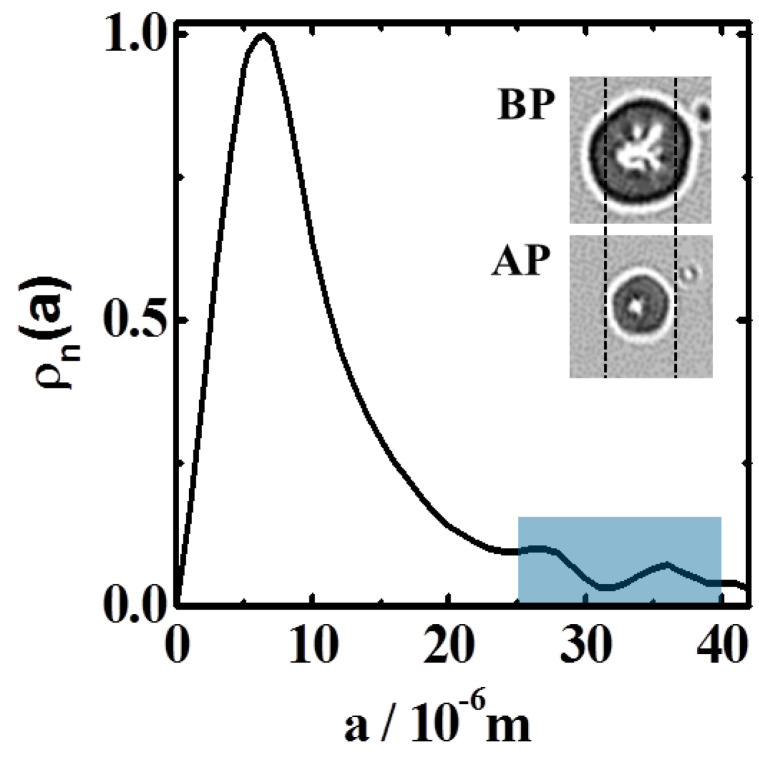
Particle number density distribution. Particles for experiments were selected from the right curve tail (marked rectangle). Pictures on the right inset (optical microscopy) illustrate the particle slimming caused by the application of plasma before (BP) and after plasma (AP). Dashed lines are visual references to observe the effect of mass removal.

Figure 1 shows the particle size-distribution resulting from gel synthesis. The number density distribution, ρ_N_, was determined automatically using ImageJ software (version number 1.51p) after proper calibration of the images. Sizes spread within a range of 1–45 μm, with the maximum located at about 6 μm. Large particles in the range 25–45 μm were selected for the experiments. This guarantees proper observation by optical microscopy after plasma application. Microscope inspection of treated particles was performed before experiments. Heating and cooling cycles showed the expected reversible sharp swollen-to-collapsed transition at 32 °C, without any significant hysteresis. This assessed the thermo-activity of the polymer after plasma application as well as the effectiveness of the BIS molecules as chemical cross-linker.

## 4. Results and Discussion

### 4.1. Radial Density-Distribution

The density of the polymer network was established by the amount of available reacting molecules and their diffusivities during the synthesis process. As commented in the introduction section, particle growth starts at nucleation points where the cross-linker molecules (initially at bulk) are massively consumed during the first steps. The center of the particles should then be denser than the peripheral areas. This fact brings consistency to a particle model with decreasing exponential density and spatial correlation characterized by a relaxation-length, *ξ*. The particle mass eroded in a plasma run (not the volume) is independent of the original particle size and can be calculated as $\Delta m=4\pi \int^{\;}r^{2}\rho \left(r\right)dr\;$+ *C*, with *C* being an integration constant. When a decreasing exponential-type density is considered, $\rho \left(r\right)=\rho_{0}exp\left(-\xi r\right)$, the loss of mass is expressed as $\Delta m=4\pi \rho_{0}\int^{\;}r^{2}exp\left(-\xi r\right)dr+C=-4\pi \rho_{0}exp\left(-\xi r\right)\left[\xi^{-1}r^{2}+2\xi^{-2}r+2\xi^{-3}\right]+C$. For the current case, in which the particle radius was reduced from *r_BP_* to *r_AP_* (size of particles before and after plasma), the amount of mass removed from each particle was expressed as:

(4) $$\Delta m\;=\;\left(4\pi \rho_{0}/\xi \right)exp\left(-\xi r_{BP}\right)\left(r_{BP}^{2}\;-\;r_{AP}^{2}\right)$$

**Figure 2:**
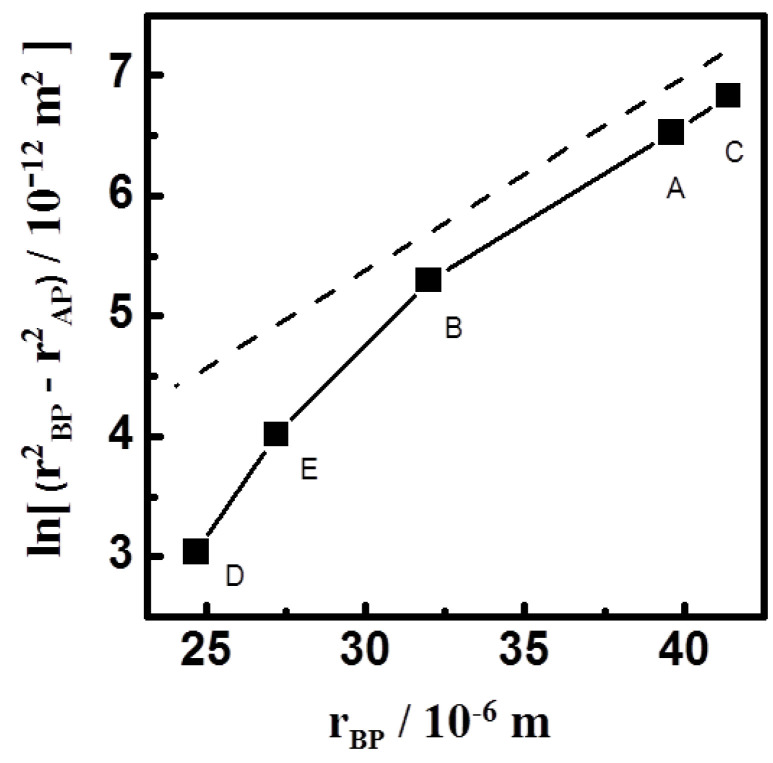
Plots of $ln\left(r_{BP}^{2}-r_{AP}^{2}\right)=ln\left(\Delta m\xi /4\pi \rho_{0}\right)+\xi r_{BP}$ as a function of $r_{BP}$, from which the density relaxation-length ξ is determined. Particles A to D used in experiments are labeled in Figure 3a.

This equation is a good approximation for large enough particles for which linear terms may be neglected. The error estimated due to the use of this approximation was below 12% for all particle-sizes employed in our experiments. Figure 2 plots the logarithm of the quadratic radii difference, $r_{BP}^{2}-r_{AP}^{2}$, as determined by optical microscopy, against the size of the original particles, $r_{BP}$. This magnitude tends asymptotically to a straight line as particle size increases, with the slope leading to a relaxation length of $\xi =\left(16.5\;\pm \;0.1\right)\times 10^{4\;}m^{-1}$. This value is common to the three largest particles (A, B and C). The density at the center was reduced with a factor *e*^−1^ at a distance outward of about $6\times 10^{-6\;}m$. It relaxed even faster for smaller particles (E and D), with larger relaxation lengths (tangent to the solid line). This result confirmed the exponential-type distribution of polymer density for our minigel particles.

### 4.2. Dynamics of Swelling

The time scale of the swelling processes was assessed by optical microscopy. Particles placed on a glass slide were dried at room temperature. Afterwards, they were gently washed with distilled water to release those not strongly adhered on the surface. Polymer swelling starts after the deposition of a drop of deionized water on the sample surface as a consequence of the hydrophilic polymer-solvent interaction present at temperatures below the pNIPAM critical temperature (≈32 °C). The solvent and room temperature were set at 20 °C, well below transition temperature. Particles evolve to their final equilibrium size by the balance between the mixing and elastic contributions to the total osmotic pressure. Size tracking was performed by a Leica DMIRB optical microscope connected to a DXC-390P Sony CCD Video Camera, with a 10 × 0.25 objective and a connection eyepiece HC 541511 (Leica) 0.5×. Spatial calibration to correct slight optical anisotropies was performed on both horizontal and vertical directions with an 80 lines/mm diffraction grating. Specific software (Section V. Alpha 4.0.3.2) analyses pictures were recorded at 30 fps. The particle-radius was finally calculated from the perimeter variation.

**Figure 3:**
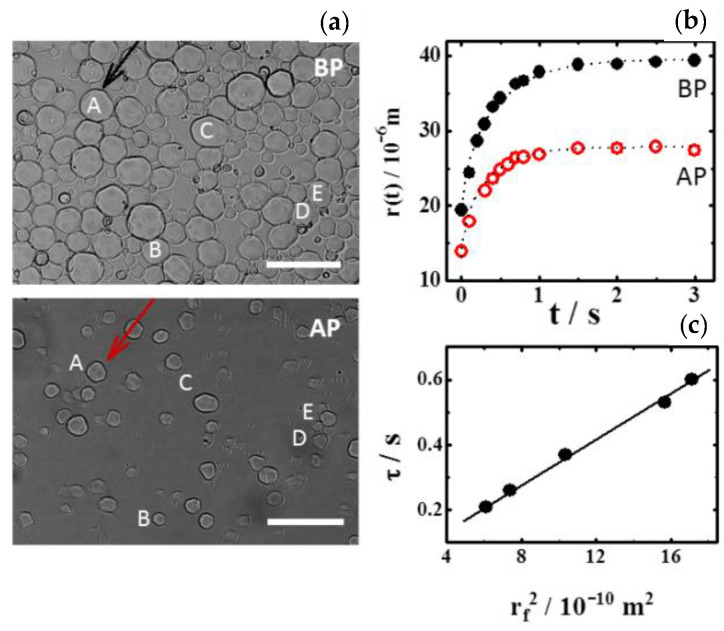
(**a**) Original (BP) and treated (AP) particles at the final swollen state. Bars correspond to 100 μm. Capital letters label the set of particles selected to characterize the radial density distribution and particle swelling dynamics. (**b**) Particle A at 20 °C tracked from collapsed to swollen state. Dotted curves are solutions of the Fick-like diffusion equation, fitted to the experimental data. (**c**) Time scale of the processes, *τ*, obtained from the fittings.

Figure 3a shows the final size after swelling for the original (BP) and treated (AP) particles selected for the experiments. Figure 3b tracks the size of the same particle A, towards the long-time equilibrium plateau, before and after the application of plasma. Due to the fast convergence of the Fourier series, five terms of equation 2 are enough for a proper process description (dotted lines). Figure 3c plots the relaxation time, *τ*, from fittings to the experimental data (particles before plasma application). This grows linearly with the particle size squared, $r_{f}^{2}$ (determined from the long-time plateau). This result is consistent with the relationship $\tau =\frac{r_{f}^{2}}{\pi^{2}}D$, reported by Tanaka’s model [11], which attributes similar diffusivity to all particles independently of their extension.

**Figure 4:**
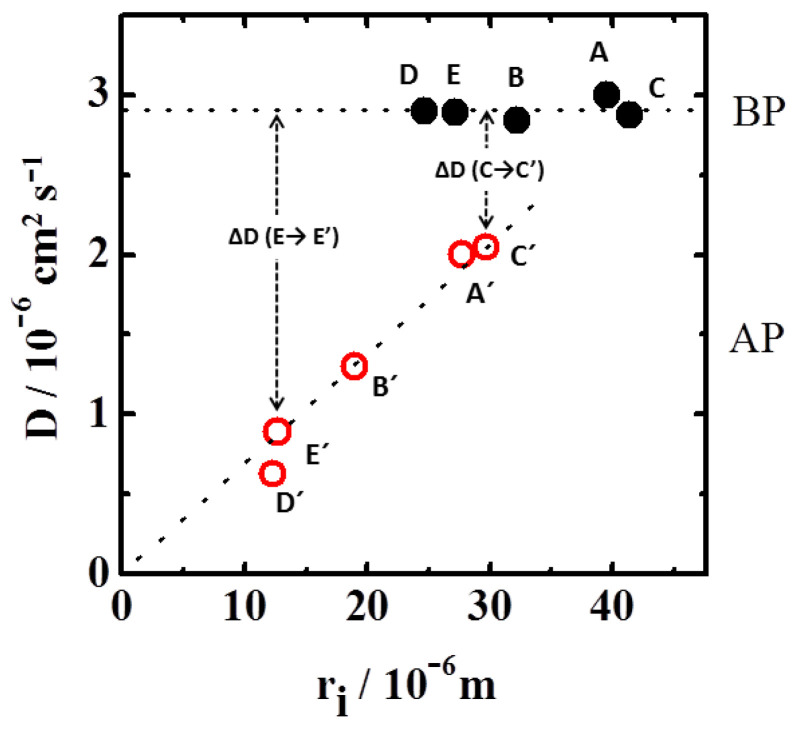
Diffusion coefficient against the particle size before (●) and after (○) plasma application. Dotted lower straight line corresponds to the linear fitting of the experimental data. *r_i_* represents the particle radius at the beginning of the swelling process.

Figure 4 plots the solvent diffusion coefficients, calculated with Equation (3), for the different particles (A, B, C, D and E) before and after plasma. Diffusion kept particle-size independent of the original particles (dark circles). However, the diffusion coefficients after plasma oxidation showed a behavior completely different (empty red circles); they were all smaller than those obtained before plasma erosion and interestingly, the diffusivities depended on particle size. The diffusivity was found to depend linearly on the particle size, $\langle D\left(r_{i}\right)\rangle =a+br_{i}\;$, with $a=\left(0.0\;\pm \;0.1\right)\times 10^{-10\;}m^{2}s^{-1}$ and $b=\left(0.07\;\pm \;0.01\right)\times 10^{-4\;}ms^{-1}$ (lower dotted line in Figure 4).

**Figure 5:**
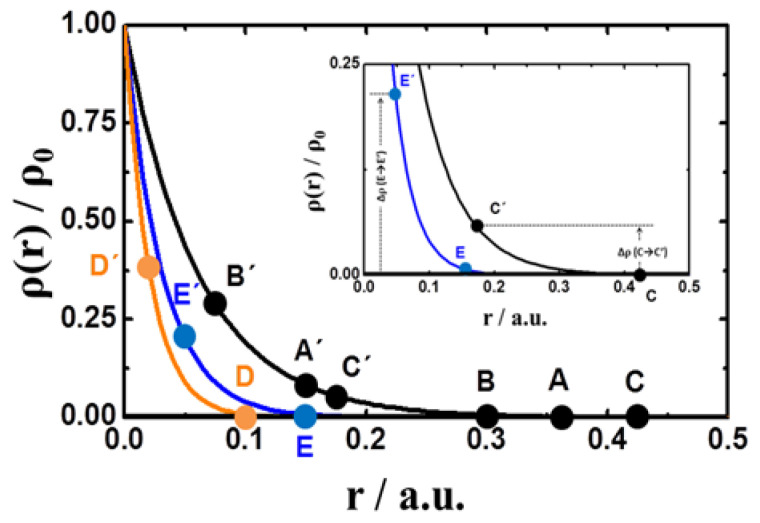
Plots of the density distribution corresponding to the set of particles used in experiments as a function of the particle radius. Capital letters without commas refer to the original particles, whereas commas on capital letters indicate the same particles after plasma application. This inset illustrates the difference in polymer density, Δρ, for particles C and E before and after plasma application.

Let us look at the particles A, B, and C before plasma application. Despite their different sizes, they exhibited similar diffusion coefficients, which is consistent with a flat polymer distribution into the particles. However, densities were not homogeneous across the particles, as shown in Figure 2. Similar diffusivities match to a scenario where the density at the particle’s borders correspond to a common flat low-density relaxation tail (as plotted in Figure 5), being the external polymer regions responsible for the main contribution to e diffusion of solvent across the polymer. Actually, the internal areas were highly cross-linked and consequently were less permeable to the solvent flow. In order to reinforce this explanation, observe that the diffusion coefficients for these three particles in Figure 4 (A, B, and C) decreased continuously after plasma size-reduction (C’, A’, B’), which is consistent with the systematic increase of density (and network compactness) along the common density curve where the new external borders will be located (C’, A’, B’ in Figure 5).

As deduced from Figure 2, particles E and D belong to different density curves (compared to A, B, and C). Since E and D exhibit the same diffusivities, and have similar diffusivities to the particles A, B and C, the borders of all particles (A, B, C, D and E) should be located at their respective low-density tails, as represented in Figure 5. Since the density distribution relaxes faster for small original particles, the density increments at the particles’ borders (after erosion) have to be larger for small original particles. See, for instance, how Δρ (E→E’) > Δρ (C→C’) in the Figure 5 inset. Consequently, the reduction of diffusivity by erosion should be greater for smaller original particles, as experimentally confirmed in Figure 4: ΔD (C→C’) < ΔD (E→E’). This scenario explains why particles with similar sizes may present different diffusivities depending on the procedure employed to reach that size. As an example, particle A’ has a similar size than particle E but exhibits much lower diffusivity. Its size comes from removing external material, thus showing a denser structure at the border, and consequently lower diffusivity.

The diffusivity of a mesh, as commented in the theoretical part of this paper, is established by the network bulk and shear moduli (*K* and μ, respectively), as well as by friction with the solvent (f). The relationship among these contributions is established by $\langle D\left(r_{i}\right)\rangle =\left[\langle K\left(r_{i}\right)\rangle +\frac{4}{3\langle \mu \left(r_{i}\right)\rangle }\right]/\;\langle f\left(r_{i}\right)\rangle$. Brackets represent average values evaluated between the beginning and the end of the swelling process. Since the mesh-density increases towards the particle center, both average values <*K*(*r_i_*)> and <*μ*(*r_i_*)> are expected to rise as the particle size reduces by plasma eroding. In addition, the friction coefficient, <*f*(*r_i_*)>, must increase proportional to the mesh-density, thus diminishing the diffusivity. A balance between all these contributions establishes the final diffusivity. Since the experiments show that the network diffusivity, <*D*(*r_i_*)>, decreases as particle size is reduced, the friction coefficient rises faster than the elastic and shear contributions.

## 5. Conclusions

Minigel particles were synthesized to study the internal density distribution and the effect of that distribution on the solvent diffusivity through the polymer network. In order to get information from the interior of the particles, the polymer located at external regions was removed by plasma etching. Higher particle densities after erosion showed internal heterogeneity, with polymer densities increasing towards the center of the particles. Decaying exponential densities were successfully tested, and the relaxation lengths estimated. The diffusion of solvent through the particles was also studied, before and after plasma oxidation. Correlation between the diffusion coefficients and the density distributions inside the particles was finally presented.

## Data Availability

The data in this study are available on request from the corresponding author.
